# Monthly Summary

**Published:** 1895-09

**Authors:** 


### Monthly Summary.

TREATING NAUSEA CAUSED BY TAKING IMPRESSIONS.—(Dr. B. Salzer).—Case 1. A lady patient, 60 years of age for whom I had extracted, with very little trouble, all the remaining upper teeth and roots, except the centrals, preparatory to making an upper set of teeth for her. I tried to take an impression in plaster of paris, but as soon as I got the impression cup into her mouth she became very much excited and inclined to nausea, which lasted about half an hour. I could not attempt it again at this sitting; at the next, I took Stent's impression compound, but even after brushing the roof of the mouth with cocaine I could not take an impression. I gave her new impression cups, together with sufficient Stent's impression compound, and told her to try herself to take an impression at her home. It was almost six months till I saw her again at my office, when she brought me two beautiful impressions, one of the upper and one of the lower. She then took another impression in my presence, and kept the compound in her mouth for almost five minutes. After the plate was made she had almost as much trouble to go through to get accustomed to wear it as she had to take an impression. Case 2. Lady, 60 years of age. Same trouble as case 1, of which I told her, and also gave her impression cups and compound, but after a short time she returned and stated that it was utterly impossible for her to accomplish it. I had in the meantime looked for something that would cure or prevent this very disagreeable nausea, and found that alcohols had been successfully used in some cases. Using pure alcohol diluted in her mouth, or giving her a little pure cognac, made a great improvement, but when I gave her a little rum it acted like magic. I could now take the impression, without the slightest trouble, with Stent's compound of plaster of paris. Before putting the finished Plate into her mouth I gave her a little rum again and had no trouble. Since the above I have only had one case to try the efficiency of the rum, and with it I had the same good results.—*Journal fur Zahnheitkunde.*

---

FILLING PULPLESS TEETH WITH FISTULOUS OPENING. —In the May number of the *Items of Interest*, Dr. C. N. Johnson, of Chicago, tells how he does this operation. The busy practitioner may not have time to resort to this proceeding, as described by Dr. Johnson. Now let me tell you how to do the operation, and do it quickly.

After getting a direct opening into the pulp chamber, and thoroughly washing out its contents with warm water from a syringe, apply the rubber dam, and dry out the pulp chamber with cotton and hot air, then with Gates-Glidden drills of different sizes, enlarge the canals to the apex; now twist a few shreds of cotton around a Donaldson broach, and saturating in pure carbolic acid and iodoform, use as a piston until the medicament appears at the fistulous opening, and do not cease until it does appear.

Now your tooth is ready to fill; do not wait a day or a week, but go right ahead and fill solidly to the apex. I use shreds of cotton saturated with chlora-percha, and a touch of iodoform, and have yet to see the first case of this kind come back to me for treatment in a practice of fifteen years.—Dr. Angus Cameron in *Dominion Dental Journal.*

---

GOLD AND PORCELAIN CROWNS.—Dr. S. W. Twilley, Baltimore, Md., describes his method of making gold and porcelain crowns similar to the Richmond system, arranged so that the porcelain fronts can be adjusted or replaced at any time. The band, cap and pin are united with solder in the usual manner. The porcelain front is then provided with a heavy backing and attached with soft wax, not bending the pins. The porcelain is then fitted to the cap and attached with hard wax, after which the porcelain is separated from the backing with a thin bladed instrument. Lead points, No. 4½, such as are used for lead pencils made by Henry Cohen, of Philadelphia, are used in the holes caused by the withdrawal of the pins, with the ends left projecting slightly. The parts are then placed in plaster and marble dust, and united with solder in the usual manner. The solder flows around the carbon points, after which the carbons are removed with a drill and the holes slightly countersunk. The crown is then set with oxiphosphate and the ends of the pins riveted.—*Dental Register.*

---

DECAYED TEETH.—Just what effect on the stomach is produced by the constant swallowing of bacteria and pus from diseased teeth, mingled with decomposing particles of food, we are unable to determine, but it is reasonable to suppose that gastric disturbances are greatly aggravated if not induced by so doing. In every community there are those who are enthusiasts on the subject of pure air and wholesome food, but whose mouths are in such a neglected condition that the air which passes through them is almost as polluted as that of crowded tenement, and every mouthful of food swallowed carries with it into the stomach millions of bacteria. The almost entire futility of sterilizing articles of diet for patients in whose mouths chronic abscesses exist, or whose teeth are covered with tartar mixed with mucus and food in a state of decomposition, need hardly be mentioned.—*Items of Interest.*

---

NEW LOCAL ANÆSTHETIC.—Dr. K. L. Schleich, of Austria, produces perfect immunity from pain by subcutaneous injections of a sugar or salt solution, or of merely cold distilled water. He adds: "The patient may remain perfectly unconscious during the amputation of hand or foot, without any of the dangers accompanying general narcosis." The phenomena is explained as follows: Local insensibility to pain is induced in the case of cocaine by purely mechanical changes, while cold water and solutions of sugar and salt act mechanically through *high paessure* and *low temperature.* Under these influences the blood and lymph are driven from the places operated upon to where the pressure is less. The tissues are thus deprived of their supply of blood, and temporary paralysis of the nerves results.—*Medical Age.*

---

MEDICINE AND DENTISTRY.—Dentistry having been respected as a specialty of medicine by all professions and the public by its thorough courses of college education, therefore we must assume that right, rather than assert it.

We can best accomplish that result by keeping strictly within our specialty and never reaching beyond, and so demand and expect the same of other specialties and practice.

Patients having systemic causes for local dental maladies should receive treatment from physicians, and then special treatment from dentists. Dentists too often prescribe systemic medicine and drugs which come under medical practice, for the administration or injection of any medicine which produces systemic effect should be attended to by the patient's physician, and that administration and operation performed in the physician's office if possible, thereby recognizing the physician's ability and decreasing the dentist's responsibility. No physician will administer anæsthetics and perform operations without an assistant, and they often criticise dentists who will do this, which without doubt is good criticism and which cannot receive too much thought from a dental standpoint.

Asserting their code of ethics and respecting it will be of profit to dentists as well as if applied to dental fraternity, that is, when patients come from other dentists with grievances, the patients having become dissatisfied, the dentist should ask them to release themselves from the former dentist, and find no fault if possible.

Often physicians send patients to dentists for examination; inquiries should then be made as to their diagnosis, and should you not agree with the physician, it is not wise to tell the patient until you have consulted him and thoroughly showed respect for his profession, which he will appreciate; and should he be correct, inform him also of progress in the case, and forget not that he will be interested.

A dentist should not deal in or sell any preparation or material if he does not intend carrying on business in preference to his profession. Should he desire any specialty, he may ask and see that the business man best suited for such work is engaged, and that specialist will respect and realize that the dentists are alive to their interests.

The theory and practice of dentistry, so little taught to physicians and druggists, should be dwelt upon at every opportunity. The public will respect the profession of dentistry in proportion as the professions of medicine, pharmacy, dentistry and the business men show respect to each other in keeping strictly within their vocation, thereby advancing the interests of each other. The greatest influence we will ever receive is that from our own professional associations and from which we will achieve the greatest benefit to success.—S. J. Renz in *Western Dental Journal.*

HYGIENE OF THE EYES.—Dr. L. W. Fox, professor of ophthalmology, Medico-Chirurgical College, Philadelphia formulates the following rules for the care of the eyes:

1. Avoid sudden changes from dark to brilliant light.

2. Avoid the use of stimulants and drugs which affec the nervous system.

3. Avoid reading when lying down, or when mentally or physically exhausted.

4. When the eyes feel tired, rest them by looking at objects at a long distance.

5. Pay special attention to the hygiene of the body, for that which tends to promote the general health acts beneficially upon the eye.

6. Up to forty years of age, bathe the eyes twice daily in cold water.

7. After fifty, bathe the eyes morning and evening with water so hot that you wonder how you stand it; follow this with cold water, that will make them glow with warmth.

8. Old persons should avoid reading much by artificial light, be guarded as to diet, and avoid sitting up late at night.

9. Do not depend on your own judgment in selecting spectacles.

10. Do not give up in despair when you are informed that a cataract is developing; remember that in these days of advancing surgery it can be removed with little danger to the vision. The same author contributes an article of some length to the November *Dietetic and Hygienic Gazette.*—*Western Dental Journal*

---

INFECTED ROOT CANALS.—Dr. De Marion, of Paris, France, has treated a large number of infected root-canals by filling them with cotton saturated with a 33 per cent. solution of formal (essentially the same as formalin), hermeticaliy sealed in, and in no instance as yet has there been any subseqent infammatory action.—*Items of Interest.*

DISINFECTANTS.—The diluting of disinfectants with alcohol, glycerine and oil makes them ineffectual. Dr. Lenti, of the Hygienic Institute of Naples, has found that corrosive sublimate dissolved in alcohol has proved useless even in 1 in 250 solution on spores which were placed in solution for 48 hours, their virulence was only weakened. By adding 10 per cent. water to the alcohol the germs were destroyed in a 1-1000 solution. A 2 per cent. solution of corrosive sublimate in pure glycerine was useless even after subjecting the spore to it for four days. By adding 40 per cent. water they were destroyed in a solution of 2-1000 in 24 hours. A 10 per cent. solution of carbolic acid in alcohol is useless, and remains so even up to 50 per cent. By adding 80 per cent. water the germs were destroyed in 48 hours. A 10 per cent. solution of carbolic acid in glycerine proved ineffectual even after 72 hours; 10 per cent. water added did not change it, but after 80 per cent. water was added it destroyed the germs in 48 hours. A 20 per cent. solution of carbolic acid in oil, and a 10 per cent. solution of lysol in oil, are both useless.—*Zahntechniche Reform.*

---

HOW TO ARREST A BOIL, CARBUNCLE, OR MALIGNANT PUSTULE.—Dr. Baker writes to the *Medical Summary* that he has used the following procedure for several years with unvarying success: Take a large hypodermic syringe, holding, say, half an ounce, fitted with a small needle. Fill it with a 1 to 500 solution of mercuric chlorid, insert the needle in one of the peripheral openings, in case it is a carbuncle, and wash out the little cavity. Then direct the needle toward and in the surrounding induration, and force a little of the solution in it. Treat each opening and its corresponding peripheral circumference in the same manner, carefully washing out the necrosed connective and other tissues that have become separated. Repeat this daily with the solution, gradually reduced to one-half the original strength, till all induration has disappeared and granulations have begun to appear. If the first injection be thoroughly performed, the spread of the carbuncle will be arrested at once, and there will be no more pain. Washing out the little cavities is painless, but the injection in the indurated tissues is not free from pain. The same treatment is applicable to the little feruncles that invade the *meatus auditorius externus* and the inner surface of the *ale nasi.—Med. and Surg. Reporter.*

---

I have observed more failures (recurrences) at this point (cusp angles) than at any other point.

This is caused largely by the former practice of leaving these angles intact, with the view of giving some support to the filling, which I believe to be a fallacy. When but little dentine is left it is entirely inadequate to sustain the easily cleaved enamel under the wear and tear of mastication.

Anchorage gives better access to the cavity margins and all parts of the cavity likewise, than the older forms.

Outward bevel all around the cavity margin (not too great) and the cavity is ready for the filling.

We go to our meetings, and we talk and talk about how to prepare cavities and introduce fillings to make them last. Now what more is there to it than the putting of all the material you can in a good, clean, properly prepared hole ?

However simple the general principles may seem, experience teaches there is a wide difference as to how the hole is cleaned and the material is put in—whether the results are to be permanent or temporary; comfortable or painful—whether put in by the master or by the apprentice, the accomplished operator or the bungler.

The cervical margin ! Place your filling material evenly and firmly on all margins and surfaces alike, maintain as uniform density as possible through the whole mass of the filling.

Restore the lost form of the tooth caused by decay and excavation. The cervical margin should now be able to take care of itself.

Amalgam is to take rank, on its merits, as a permanent filling material, both for rich and poor; for resisting decay; for easier form-building; and for its adaptability to the cervical margin.—*Review*.

---

THE TREATMENT OF EPITHELIOMA.—This is the patient upon whom we operated some time ago for an epithelioma on the right side of the chin, extending almost to the vermillion border of the lower lip. Under a general anæsthetic, I cut away all the rank tissue, as deep as the periosteum of the lower jaw, but as it was uncertain whether the bone was involved in the malignant process or not, I did not remove the inferior maxilla. The bone was somewhat roughened, but there were no signs of granulation tissue, such as we see in malignant bone disease. I scraped the wound thoroughly and then applied Marsden's paste, which has the faculty of destroying only diseased tissue; it does not destroy healthy tissue. This paste destroyed the superficial layer of the bone, proving that in spite of its healthy appearance, it was already involved by the disease to some extent.

In St. Louis, on the third of this month, I delivered an address on surgery, which consisted of a summary of those points in surgical work which attracted my attention most during the past year. One of the points brought up for discussion was the treatment of superficial epithelioma. On this subject I have undergone a decided change of mind during the past five or six years. If I had a superficial epithelioma develop anywhere on my body where I could use Marsden's paste, I would prefer that method of treatment to the knife. In cases where the disease has existed for so long a period that the paste alone cannot be relied upon, I would prefer to have the malignant process first cut or scraped away, and then have the paste applied. In this way we get more satisfactory results than by any other treatment I know of.

The formula for Marsden's paste, which I have given you a number of times, is as follows:—

| | | |
|---|---|---|
| Acid arsenious | - - | 2 drachms. |
| Pulv. gum arabic | - - | 1 drachm. |
| Cocaine muriate | - - | 18 grains. |

When you are ready to use this it should be made into a paste by adding water. The paste should be of the consistency of rich cream and should be applied to the wound on a small piece of cloth, which is left on from eighteen to thirty-six hours. This can be repeated as often as is necessary. The above is the formula for the stronger paste. In the weaker preparation we use only one drachm of arsenious acid and twelve grains of cocaine. Marsden's original formula consisted only of equal parts of arsenious acid and gum arabic. The cocaine has been added to counteract the pain; it has never, to my knowledge, given rise to constitutional symptoms.

It is a fact that arsenious acid, in certain combinations with gum arabic, forms a most potent remedy in the treatment of malignant disease. In many cases coming under my own observation its use has been followed by most satisfactory results, effecting a complete cure and no recurrence taking place. It is difficult to say whether the action of the remedy is directly mechanical and partially phagocytic, which practically means that one bug eats up another bug. It is possible that by these applications a suppurative process is set up, the germs of which may eat up the rank tissue of the malignant disease, or kill the specific germ which causes such disease. Epithelioma is probably produced by some germ, although it has not yet been discovered. Following the law of analogy, it is a case of the survival of the fittest, which runs through all the universe. The theory of phagocytosis has been actually proven in connection with the white blood corpuscles, which have the power to destroy certain bacilli.

The same theory applies in the treatment of sarcoma with the toxines of erysipelas. I have seen several cases of undoubted sarcoma which were cured after accidental inoculation with the erysipelas cocci. In one of these cases, a man with a large sarcoma of the abdomen, ten or twelve years have elapsed and there has been no recurrence. In several other cases, five or six years have elapsed.

To show the value of Marsden's paste, the following case is of interest: A boy, about twelve years of age, developed a tumor on the forehead. His physician, who regarded it as an ordinary sebaceous cyst, cut it out. It returned within six or eight weeks. I then saw the boy, and recognizing the growth as a malignant one, I performed a very radical operation, cutting away all the tissues down to the temporal fossa. It recurred within four months. Dr. A. R. Robinson then saw the case with me in consultation and we decided to apply Marsden's paste. We did so and the result was entirely satisfactory. This was many years ago, and up to the present time there has been no recurrence, nor is there likely to be.—*International Journal of Surgery.*

---

FOR HEMORRHAGE.—Dr. Blaschko, of Berlin, uses a solution of one pint each of gallio acid and ergotini in a mixture of 25 parts each of distilled water and syrup of althæa, of which a teaspoonful is to be taken every few h urs. —*Deutsche Medizenal Zeitung.*

---

METALLURGY—Dr. D. B. McHenry, Grenada, Miss., said before the Mississippi Dental Association, "We believe we should return to the earlier methods in prosthetic dentistry, before the advent of rubber plates, by the abuse of which the profession has been 'abused, vilified, degraded to the lowest depths at the hands of charlatans.'"

